# Public Medical Preparedness at the “Swiss Wrestling and Alpine Games 2013”: Descriptive Analysis of 1,533 Patients Treated at the Largest 3-Day Sporting Event in Switzerland

**DOI:** 10.1155/2017/9162095

**Published:** 2017-02-06

**Authors:** Simone Hostettler-Blunier, Nora Müller, Tobias Haltmeier, Andreas Hosner, Heinz Bähler, Frank Neff, Daniel Baumberger, Aristomenis Exadaktylos, Beat Schnüriger

**Affiliations:** ^1^Department of Emergency Medicine, Bern University Hospital, Bern, Switzerland; ^2^Sanitätspolizei Bern (Emergency Medical Service), Bern, Switzerland; ^3^Department for Visceral Surgery and Medicine, Division of Acute Care Surgery, Bern University Hospital, Bern, Switzerland; ^4^Swiss Wrestling and Alpine Games (SWAG), Burgdorf, Switzerland

## Abstract

*Introduction*. Medical preparedness at mass gatherings is challenging, as little is known about the optimal planning. Most studies and case reports are based on mass casualty incidents, so the results cannot be extrapolated to mass gatherings. Aim of this study was to evaluate the preclinical medical structure and the frequency of specific injuries and medical emergencies during the event.* Methods*. Retrospective analysis of a prospectively collected database. Three on-site medical assistance points were set up, completed by mobile teams, and coordinated by an on-site operational management team. Medical staff requirements were calculated using Maurer's formula.* Results*. A total of 1,533 patients were treated during the three-day event. Overall, the medical usage rate (MUR; patients per 10,000 visitors) was 51.1. A total of 58 patients (3.8%) required a hospital transfer. In 1,063 cases (69.3%) a diagnosis was documented. Of these, 503 patients (47.3%) suffered from hymenoptera stings; the two most common non-trauma-related diagnoses were alcohol/drug intoxication (4.1%) and gastrointestinal diseases (4.0%).* Conclusion*. Overall, the on-site medical care worked well. However, a high frequency of hymenoptera stings occurred, resulting in a shortage of antihistamine medication. Moreover, more than half of the patients were managed at the second largest medical assistance point. Prospective and critical evaluation of medical care at mass gatherings is crucial in order to optimize on-site medical preparedness at future events.

## 1. Introduction

Medical preparedness at mass gatherings is challenging as many different eventualities have to be considered. Whereas multiple reports on mass casualty incidents are available, literature on the medical care as well as patient characteristics at regular mass gatherings is scarce. Moreover, lessons learned from mass casualty reports may not be applicable to mass gatherings, such as sporting or musical events.

Locoh-Donou et al. in a retrospective review evaluated 79 different mass-gathering events, including sporting and nonsporting events [1]. A total of 670 patients presented at these events including mostly young people with the highest number of cases in sport-events such as football matches. The overall medical usage rate (MUR; patients per 10,000 visitors) was 7.9.

Furthermore, there are two reports about the prehospital care of spectators during the 2004 Summer Olympic Games in Athens [2] and the 2002 Olympic Winter Games in Salt Lake City [3]. During the 2004 Olympic Games in Athens, 10,564 people required medical aid, half of which due to orthopaedic injuries. The MUR was not calculated in this report. During the 2002 Olympic Winter Games an overall spectator MUR of 26.9 was calculated. The most common diagnoses were miscellaneous injuries such as lacerations, contusions/abrasions, and spine injuries.

Due to the difficulty in prospectively collecting detailed patient characteristics treated at mass gatherings, little data is available to allow an optimal planning of medical care at these events. The aim of this study was, therefore, to evaluate the planning of the on-site medical care during a three-day mass gathering, the “Swiss Wrestling and Alpine Games” (SWAG) 2013, using a prospectively collected database.

## 2. Methods

### 2.1. Study Design

This observational study assessed the medical care at a large mass gathering in Switzerland, the Swiss Wrestling and Alpine Games taking into account medical resources and staff, number of visitors, and patient and injury characteristics.

During the entire three event days, patient and injury characteristics of patients treated at one of the three medical assistant points were prospectively collected by the medical staff on site. Subsequently, de-identified patient data was further analyzed at the Bern University Hospital. The medical staff on site included physicians, fully trained paramedics, and medics of the Swiss Armed Forces trained in advanced first aid and Samaritans trained in basic first aid medicine. Patients were classified according the National Advisory Committee for Aeronautics (NACA) score [4, 5] by a fully trained paramedic or attending physician.

The following variables were prospectively collected:

*Patients with NACA scores 0-1*: date of birth, gender, time of admission and discharge from the medical assistant point, and diagnosis.
*Patients with NACA scores >1*: date of birth, gender, time of admission and discharge from the medical assistant point, diagnosis, therapeutic measures, monitoring of vital signs, hospital transfer, and mode of transportation. Medical conditions were grouped into 17 categories. When multiple diseases existed, the most severe was considered.

Categorical variables were expressed as number and percentages and continuous variable as mean and standard deviation (SD).

### 2.2. On-Site Management of Medical Care

Medical preparedness was organized and simulated by the Bern Emergency Medical Service (EMS, “Sanitätspolizei”), Police, and Fire Department, together with the Federal Office for Civil Protection (“Bundesamt für Bevölkerungsschutz”). The following scenarios were considered: fire and explosions, mass casualty of injured patients, bomb threat, panic or mass movements, flood, severe criminal offense, technical problems (e.g., grandstand collapse and power failure), and poisoning by gas.

The civil EMS coordinated the medical care on site, together with the Swiss Armed Forces and Swiss civil defense, that supported the EMS with medically trained personnel and equipment. The on-site operational management team comprised two senior staff members of the Bern EMS and one senior staff member of civil defense. This on-site operational management team was located directly on the festival ground and communicated closely with the medical assistant points and hospitals if necessary.

**Figure 1:**
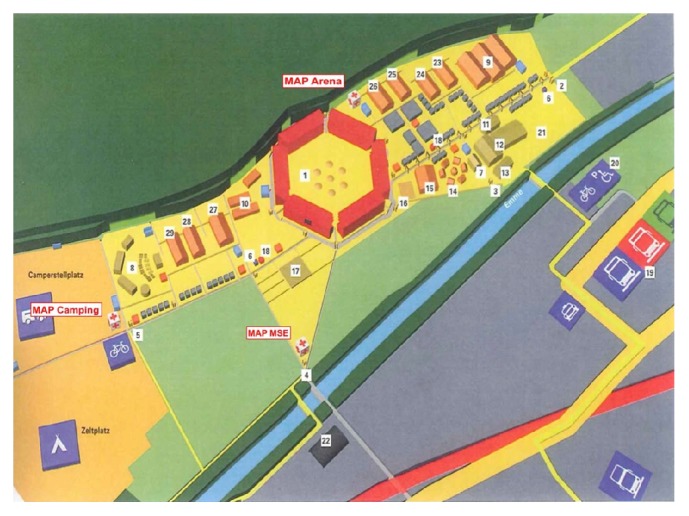
Overview of the event site and medical assistant points (MAP).

**Figure 2:**
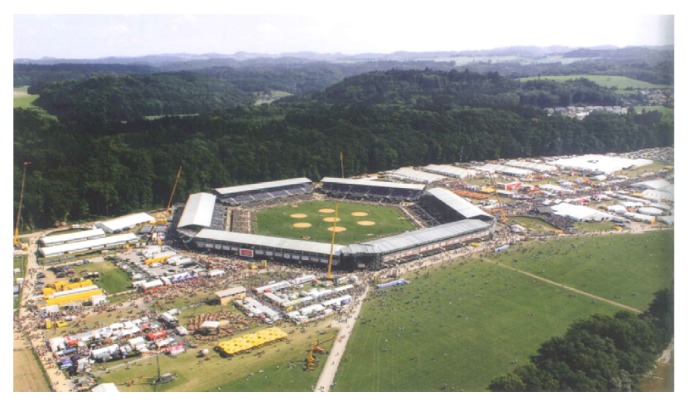
Overview over the main part of the venue.

A total of three medical assistant points were established throughout the festival grounds: ARENA, MSE 2 (“Modulare Sanitätseinheit”), and CAMPING (Figures 1 and 2). In addition, there were 19 mobile medical teams comprising two paramedics at the tribunes nearby the sports fields, as these were the areas with the highest density of visitors. The mobile teams were responsible for the initial medical care. Subsequently, patients were brought to one of the three medical assistant points for further treatment if necessary.

In case patients required hospital transfer, two EMS teams were available for ground transportation at all time. Patients were primarily transferred to a regional hospital. This hospital is staffed with emergency physicians, anesthesiologists, trauma surgeons, and provides operating rooms, computed tomography scanning, and magnetic resonance imaging. The closest Level I Trauma Center was the Bern University Hospital, which is located 25 km away from the festival ground.

#### 2.2.1. Medical Assistant Point ARENA

The location of the medical assistant point ARENA was chosen based on its proximity to an area with an anticipated high density of visitors, that is, the arena. The medical supply was provided by the Bern EMS and replenished overnight. The patients were evaluated and treated by physicians or paramedics.

The ARENA included a total of 10 beds. In patients with severe medical conditions, the planned time from admission to ARENA to hospital transfer was 30 minutes. Overall, ARENA was operated by 25 Samaritans, 3 paramedics, and 2 physicians. From 07:00 h to 18:00 h there were 13 Samaritans, 2 paramedics, and one physician present. At night (18:00 h–04:00 h) there were 12 Samaritans, one paramedic, and one physician on site.

#### 2.2.2. Medical Assistant Point MSE 2 (“Modulare Sanitätseinheit”)

The medical assistant point MSE 2 was operated and equipped by the Swiss Armed Forces according to the “Reglement 59.021 d (Sanitätsdienst der Truppen)”. Its location was chosen in order to facilitate and secure rescue and fast hospital transportation. The MSE 2 provided a total of 20 beds and was staffed during the day (07:00 h–19:00 h) with 12 medics of the Swiss Armed Forces, 5 Samaritans, 2 paramedics, and 1 physician. At night (19:00 h–07:00 h) it was operated with only one Samaritan less than at daytime.

#### 2.2.3. Medical Assistant Point CAMPING

The medical assistant point CAMPING was the smallest of the three medical assistance points, providing 2 beds. It was managed by 4 Samaritans and 2 paramedics during the day (07:00 h–19:00 h) and 4 Samaritans and 1 paramedic at nighttime (19:00 h–07:00 h), without the presence of a physician. It was provided with less medical equipment compared to the ARENA and MSE 2. Each patient with major injuries had to be reported to the on-site operational management team, who decided whether the patient required transfer to one of the other two medical assistant points or to a hospital.

#### 2.2.4. Personnel Planning

Planning of the medical equipment, medical staff, and their duties was carried out by the Bern EMS. The required medical staff was calculated using Maurer's formula [6].

Maurer's formula was developed to estimate the hazard potential of large events. The formula gives the number of medical staff needed, taking into account the estimated number of visitors (points-based system), the type of the event, and special circumstances, such the attendance of very important people or violent groups (multiplicators).

For the SWAG 2013, the organizing committee anticipated different numbers of visitors at each day of the event. The number of visitors was estimated using data from past SWAG events. The expected number of visitors was 80,000 at day one, 100,000 at day two, and 120,000 at day three. Based on these numbers the calculated number of required medical staff was 70 at days 1 and 2 and 119 on day 3, respectively.

### 2.3. Statistics

This was a purely descriptive analysis; no statistical data comparison was performed. Data collection was performed using Microsoft Excel (Microsoft Corporation, Redmond, WA, USA).

## 3. Results

During daytime, an average of 50 Samaritans, 37 paramedics, 12 members of the Swiss Armed Forces, and 7 physicians were on site. During the nights, there were 36 Samaritans, 16 paramedics, 12 members of the Army, and 4 physicians present on average. Overall, the 468 members of the medical staff (Samaritans, paramedics, members of the Swiss Armed Forces, and physicians) were present on site during a total of 5,399 hours.

### 3.1. Demographics and Characteristics of Patients

A total of 1,533 patients were treated at one of the three medical assistant points, corresponding to an overall MUR of 51.1 (1,533 per 300,000 visitors). Mean age of patients was 37.3 (SD 16.7) years.

A total of 242 patients (15.8%) were treated on day 1, 863 patients (56.3%) on day 2, and 428 patients (27.9%) on day 3. Overall, 811 patients (52.9%), were treated at ARENA, 321 patients (20.9%) at MSE 2, and 401 patients (26.2%) at CAMPING.

**Figure 3:**
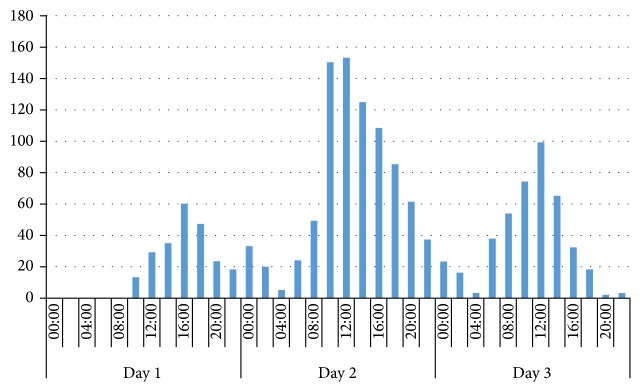
Chronological distribution of patients treated.

On all three festival days, the frequency of treatments peaked between 12 a.m. and 4 p.m. (Figure 3). The highest frequency of admissions was observed on day 2 between 12 a.m. and 4 p.m. During this time, 153 patients were admitted to one of the three medical assistant points.

Mean time spent within the medical assistant points was 12.5 (SD 26.6) minutes per patient. A total of 127 patients (11.9%) were treated longer than 30 minutes (mean treatment time 76.1 [SD 55.0] min). Of these, 108 patients (85.1%) had an NACA score >1, including 30 patients with acute alcohol intoxication.

A total of 58 patients (3.8%) required hospital referral for further treatment. The most common medical emergency requiring hospital admission was musculoskeletal injuries to the ankle (*n* = 10; 17.2%) or to the forearm (*n* = 4; 6.9%). Injuries that required hospital admission are summarized in Table 1.

Table 2 shows an overview of medical emergencies treated during the three festival days. The most commonly treated medical emergency was hymenoptera stings (*n* = 503 patients, 47.3%). Of these, 71 (14.1%) were potentially life-threatening stings, including stings to the face, enoral stings, and stings in patients with known allergies. Nine patients (1.8%) suffering from hymenoptera stings required hospital referral due to stings to the lips or enorally.

A total of 98 patients (9.2%) presented with musculoskeletal injuries. Of these, 60 patients (61.2%) suffered serious musculoskeletal injuries (suspected diagnosis of fracture or luxation), most frequently to the lower extremities (*n* = 23; 38.3%). Hospital transfer for further treatment was required in 21 of these patients. All other diagnoses were found in less than 5% of cases (Table 1).

Overall, 63 patients (5.0%) were <16 years of age. Of these younger patients, 50 patients (79.3%) had a documented diagnosis (39 wasp and bee stings, 6 small wounds, and 2 large wounds, 2 otolaryngological disease cases, and 1 musculoskeletal injury).

### 3.2. Patients with NACA Scores 0-1

Of the total of 1,063 patients with a documented diagnosis, 789 patients (74.2%) were treated for injuries with a NACA score of 0-1. This group comprised 435 (55.1%) male and 304 (38.5%) female patients. Mean age was 37.4 years (SD 14.9). Hymenoptera stings accounted for 432 (54.7%) and small wounds for 186 (23.6%) of all documented minor injuries. Most small wounds were blisters and abrasions.

### 3.3. Patients with NACA Scores >1

A total of 274 patients (25.8%) suffered from injuries corresponding to a NACA score >1. In this group of patients mean age was 39.2 years (SD 17.6) and 182 patients (66.4%) were male. Five patients (1.8%) were younger than 16 years old. The most frequent severe injuries were hymenoptera stings (*n* = 71, 25.9%) at critical locations (i.e., the face, tongue, or neck) or in patients with known allergy. These were followed by musculoskeletal injuries (*n* = 60, 21.9%) and alcohol or drug intoxications (*n* = 32, 11.7%). Neurological diseases (migraine, suspicion of stroke, and somatosensory disorders) were found in 15 patients (5.5%), wounds requiring suturing in 14 patients (5.1%), and gastrointestinal disease in 14 patients (5.1%).

## 4. Discussion

At the SWAG 2013, which is the largest three-day sporting event in Switzerland taking place every 3 years, prehospital medical care was provided in three medical assistant points and by nineteen mobile medical teams. Out of the 300,000 visitors [7], 1,533 required medical care (MUR 51.1). In 1,063 patients (69.3%) a diagnosis was documented on site. A total of 789 patients (74.3%) suffered from injuries corresponding to a NACA score of 0-1 and 274 patients (25.8%) presented with a NACA score >1. Fifty-eight patients (5.5%) required hospital transfer for further treatment.

The number of patients treated at the SWAG 2013 is impressive when compared to the number of patients treated on a weekend during summer at the Emergency Department at Bern University Hospital (approximately 400 patients in three days).

The World Health Organization defines mass gatherings as organized, special events in which the number of visitors strains or overwhelms the planning and response resources of the community hosting the event [8]. At events with more than 1,500 participants, a duration longer than three hours, and transport time to the next emergency medical care facility longer than 10 minutes, as well as events that are associated with an increased risk for injuries or the attendance of specific risk groups, on-site professional medical staff is recommended [9].

Of note, in order to estimate the total number of required health professionals, the calculated number of health professionals according to Maurer's formula was multiplied with the number of planned work shifts. Taking this into account, the estimated number of medical staff met the effective number required at the SWAG.

More than 50% of patients were treated in the second largest medical assistance point ARENA, most likely due to its proximity to the main festival area with a high density of visitors (Figure 1).

Only one-fifth of patients were treated at the medical assistant point MSE 2, which was the largest of the three medical assistant points. At the smallest medical point CAMPING 37.7% of all patients were treated, again more than at the medical point MSE 2. However, at the medical point CAMPING, only patients with minor injuries (NACA score 0-1) were treated. Although the treatment was longer than the estimated time of 30 minutes in 12% of patients, none of the medical assistant points reported an overflow of patients.

Of all 1,533 patients treated during the three festival days, a diagnosis was documented in 1,063 patients (69.3%). Although this is a reasonable number of patients with a specific diagnosis when taking into account that medical care was performed on scene, there is still a high proportion of patients with no documented diagnosis, which is a major limitation of this study. More accurate and complete documentation of diagnoses during future mass gatherings is of paramount importance in order to provide reliable data for the planning of upcoming similar events.

Injury characteristics and medical emergencies during mass gatherings as well as the MUR depend on several factors. According to the literature, the MUR ranges between 5 and 185 patients per 10,000 attendees [2, 10, 11]. This wide range is explained by the different types of events that were assessed (rock concerts, sporting events, political events, etc.). The different number of visitors or participants, bounded versus unbounded venue, event duration, consumption of alcohol or illegal drugs, characteristics of attendees, weather conditions, and the terrain all have an impact on the required medical staff, making an exact calculation and planning extremely difficult [10, 12–15]. Again, the exact documentation of patient characteristics would greatly improve the planning of future events.

With 51.1, the MUR was relatively high at the SWAG 2013 compared to the literature, mainly due to the high frequency of hymenoptera stings. The high frequency of stings, in turn, may be explained by the good weather conditions and high temperatures as well as the rural location of the festival ground (Figure 2). The high number of patients with hymenoptera stings led to a shortage of antihistamine medication, which was the only reported incorrect planning at this event.

## 5. Conclusion

In general, the on-site medical care at the SWAG 2013 worked well. The medical operational management team on site may have avoided an overflow of patients, even though the distribution of health professionals at the three medical assistant points did not match the number of patients that presented at the medical assistant points during the event: the second largest medical assistant point was confronted with the largest number of patients. It is important to prospectively and critically evaluate prehospital health care during mass gatherings in order to optimize on-site medical preparedness for similar future events.

## Figures and Tables

**Table 1:** Medical conditions requiring hospital referral.

	Day 1		Day 2			Day 3		Total	%
	MSE 2	ARENA	MSE 2	ARENA	Child	MSE 2	ARENA		
Musculoskeletal	2		8	4		4	3	21	36.2
Stings			1	7			2	10	17.3
Neurological				4	1	1		6	10.4
Wounds large			1	2		2		5	8.6
Otolaryngological			1	2		1		4	6.9
Gastrointestinal	1		1				1	3	5.2
General disease				2				2	3.4
Alcohol/drugs		1	1					2	1.7
Skin						1		1	1.7
Ophthalmological							1	1	1.7
Heart				1				1	1.7
Lungs					1			1	1.7
Burns large			1					1	1.7

*Total*	*3*	*1*	*14*	*22*	*2*	*9*	*7*	*58*	*100*

**Table 2:** Medical emergencies during the three festival days and different medical assistant point.

	Day 1			Day 2			Day 3			Total	%
	MSE 2	ARENA	Camping	MSE 2	ARENA	Camping	MSE 2	ARENA	Camping		
Stings	27	6	36	59	134	94	21	98	28	503	47.3
Wounds small	10	6	38	12	30	57	10	25	12	200	18.8
Musculoskeletal	4	1		19	22	12	16	21	3	98	9.2
Alcohol/drugs		4		9	7	12	7	5		44	4.1
Gastrointestinal	1	1		7	10	2	4	16	1	42	4.0
Wounds large	3	1		13	2	3	11	3		36	3.4
Otolaryngolocial	1		1	3	8	11	1	2	5	32	3.0
Headache	2			12	3	2	2		5	26	2.5
General disease	2		2	4	4	1	2	2		17	1.6
Neurological				1	7	1	3	3		15	1.4
Ophthalmological	1			5	3		1	4		14	1.3
Skin			1	3		1	2	4		11	1.0
Burns small			1	2	1	4				8	0.7
Lungs				1	1			1	2	5	0.5
Heart	1				2		1	1		5	0.5
Urological				2			2	1		5	0.5
Burns large				2						2	0.2

*Total*	*52*	*19*	*79*	*154*	*234*	*200*	*81*	*186*	*56*	*1063*	*100*

## References

[1] Locoh-Donou S., Guofen Y., Welcher M., Berry T., O'Connor R. E., Brady W. J. (2013). Mass-gathering medicine: a descriptive analysis of a range of mass-gathering event types. *American Journal of Emergency Medicine*.

[2] Tsouros A. D., Efstathiou P. A. (2007). *Mass Gatherings and Public Health—The Experience of the Athens 2004 Olympic Games*.

[3] Allen T. L., Jolley S. J., Cooley V. J. (2006). The epidemiology of illness and injury at the alpine venues during the Salt Lake City 2002 Winter Olympic Games. *The Journal of Emergency Medicine*.

[4] Tryba M. B., Echtemeyer V. (1980). Klassifizierung von Erkrankungen und Verletzungen im Notarztrettungssystem. *Notfallmedizin*.

[5] Weiss M., Bernoulli L., Zollinger A. (2001). The NACA-scale. Construct and predictive validity of the NACA-scale for prehospital severity rating in trauma patients. *Der Anaesthesist*.

[6] Maurer K. (2001). *Einsatzplanung bei Grossveranstaltungen. Handbuch für Schnelleinsatzkräfte*.

[7] “Eidgenössisches” 2013: eine Erfolgsgeschichte, 2014 [Medienmitteilung], http://www.burgdorf2013.ch/cgi-bin/ckfinder/files/Medien/2014/mm_schlussbericht_d_140228.pdf

[8] WHO Organization WHO Inter-departmental Mass Gathering Groups. http://www.who.int/ihr/ith_and_mass_gatherings/mass_gatherings/en/.

[9] Soomaroo L., Murray V. (2012). Weather and environmental hazards at mass gatherings. *PLoS Currents*.

[10] Milsten A. M., Maguire B. J., Bissell R. A., Seaman K. G. (2002). Mass-gathering medical care: a review of the literature. *Prehospital and Disaster Medicine*.

[11] Carlson L. (1992). Spectator medical care: learning from the super bowl. *Physician and Sportsmedicine*.

[12] Soomaroo L., Murray V. (2012). Disasters at mass gatherings: lessons from history. *PLoS Currents*.

[13] Turris S. A., Lund A., Bowles R. R. (2014). An analysis of mass casualty incidents in the setting of mass gatherings and special events. *Disaster Medicine and Public Health Preparedness*.

[14] Grissom C. K., Finnoff J. T., Murdock D. C., Culberson J. T. (2006). Nordic venue medical services during the 2002 Winter Olympics. *The Journal of Emergency Medicine*.

[15] De Lorenzo R. A. (1997). Mass gathering medicine: a review. *Prehospital and Disaster Medicine*.

